# Long-Term Coffee Consumption is Associated with Fecal Microbial Composition in Humans

**DOI:** 10.3390/nu12051287

**Published:** 2020-05-01

**Authors:** Sonia González, Nuria Salazar, Sergio Ruiz-Saavedra, María Gómez-Martín, Clara G. de los Reyes-Gavilán, Miguel Gueimonde

**Affiliations:** 1 Department of Functional Biology, University of Oviedo, 33006 Oviedo, Spain; soniagsolares@uniovi.es (S.G.); mariagomart@gmail.com (M.G.-M.); 2 Diet, Microbiota and Health Group, Instituto de Investigación Sanitaria del Principado de Asturias (ISPA), 33011 Oviedo, Spain; nuriasg@ipla.csic.es (N.S.); sergioruizsa3@gmail.com (S.R.-S.); greyes_gavilan@ipla.csic.es (C.G.d.l.R.-G.); 3 Department of Microbiology and Biochemistry of Dairy Products, Instituto de Productos Lácteos de Asturias (IPLA-CSIC), 33300 Villaviciosa, Asturias, Spain

**Keywords:** coffee, (poly)phenol, gut microbiota, *Bacteroides*

## Abstract

Coffee consumption has been related to a preventive effect against several non-transmissible pathologies. Due to the content of this beverage in phytochemicals and minerals, it has been proposed that its impact on health may partly depend on gut microbiota modulation. Our aim was to explore the interaction among gut microbiota, fecal short chain fatty acids, and health-related parameters in 147 healthy subjects classified according to coffee consumption, to deepen the association of the role of the (poly)phenol and alkaloid content of this beverage. Food daily intake was assessed by an annual food frequency questionnaire (FFQ). Coffee consumption was categorized into three groups: non-coffee-consumers (0–3 mL/day), moderate consumers (3–45 mL/day) and high-coffee consumers (45–500 mL/day). Some relevant groups of the gut microbiota were determined by qPCR, and concentration of fecal short chain fatty acids by gas chromatography. Serum health related biomarkers were determined by standardized methods. Interestingly, a higher level of *Bacteroides–Prevotella–Porphyromonas* was observed in the high consumers of coffee, who also had lower levels of lipoperoxidation. Two groups of coffee-derived (poly)phenol, methoxyphenols and alkylphenols, and caffeine, among alkaloids, were directly associated with *Bacteroides* group levels. Thus, regular consumption of coffee appears to be associated with changes in some intestinal microbiota groups in which dietary (poly)phenol and caffeine may play a role.

## 1. Introduction

Coffee is one of the most consumed non-alcoholic beverages worldwide and it may exert different effects at a physiological level [1]. Although it has traditionally been considered as a beverage with very low nutritional value, epidemiological evidence suggests that moderate coffee consumption may reduce the risk of chronic diseases such as metabolic syndrome, obesity, type 2 diabetes [2], cardiovascular diseases [3], or some types of cancer [4,5,6]. Coffee may impact directly on the host gastrointestinal physiology by increasing intestinal motility and reducing intestinal transit time [7,8]. Some of these widely described benefits of coffee have been attributed to its high content in non-nutritional compounds such as phenolic compounds, fibers, minerals, and caffeine [9], which may also influence host metabolic pathways related to health maintenance. From these compounds, caffeine, (poly)phenols, and fibers are able to reach and exert some of their effects in the large intestine, being fermented by the gut microbiota [10]. Thus, given the pivotal role that microbiota plays on human nutrition and health [11], it is possible that some of the beneficial effects of the coffee components may be related with the participation of the gut microbiota in the metabolism of such compounds. Interventional studies analyzing the impact of a moderate coffee intake during three weeks in a healthy population have reported an increase of *Bifidobacterium* [9], sometimes also linked to a decrease of *Clostridium* and *Escherichia coli* [9,12,13,14]. Regarding other bacterial groups, such as *Bacteroides*, the results in the literature remain controversial [9,13,15,16]. Among the possible mechanisms to explain these associations, data from in vitro studies pointed to a direct relationship between chlorogenic acids and selective changes on the *Blautia coccoides–Eubacterium rectale* group [10] and between caffeine and the abundance of the *Lactobacillus* species [17]. Based on previous evidences indicating that theobromine, an alkaloid present in coffee, can enhance (poly)phenol absorption in the intestine [18], a synergistic effect for phenolic compounds and alkaloids on the intestinal microbiota at this location may be plausible. To date, most of the studies analyzing the impact of coffee on the composition of the intestinal microbiota come from in vitro, animal, or intervention studies. However, to the best of our knowledge, no observational studies are currently available analyzing the impact of regular coffee consumption on fecal microbiota, taking into consideration the influence that the content of this beverage in caffeine and phenolic compounds may exert on the microbiota. This information would contribute to expand the existing knowledge about the impact of coffee on gastrointestinal physiology and therefore, on health maintenance. 

## 2. Subjects and Methods

The study included 147 participants, with ages ranging from 19 to 95 years and body mass index (BMI) scores between 19.0 and 39.0 kg/m^2^ who were recruited in Asturias (Atlantic coast of Spain). Volunteers were cited individually, informed about the study, and gave informed written consent before enrolment. Inclusion criteria were the absence of diagnosed immune or digestive related pathologies as well as non-consumption of corticoids, immunosuppressive drugs, monoclonal antibodies, antibiotics, or immunotherapy, and not having consumed probiotics or prebiotics as dietary supplements during the previous month.

The study was approved by the Bioethics Committee of CSIC (Consejo Superior de Investigaciones Científicas) and the Regional Ethics Committee for Clinical Research (Servicio de Salud del Principado de Asturias n 13/2010).

### 2.1. Nutritional Assessment

Participants were instructed to maintain their usual dietary pattern before the study. Regular food intake was assessed by trained personnel in a personal interview of approximately 1 h duration, using an annual semi-quantitative food frequency questionnaire (FFQ), previously validated [19,20]. Methodological issues about dietary assessment were published elsewhere [18]. Food consumption was transformed into energy and macronutrients intake using the food composition tables of CESNID (Centro de Enseñanza Superior de Nutrición Humana y Dietética) [21]. Caffeine intake was estimated from the United States Department of Agriculture (USDA) food composition database [22]. The polyphenols content in foods was completed using the Phenol Explorer database that compiled detailed information from over 400 foods and beverages, including coffee [23] and fiber components, and were ascertained using the Marlett et al. food composition tables [24]. 

At the time of carrying out the blood extraction, height and weight were taken by standardized protocols previously described [25] in order to calculate the BMI by the formula: weight (kg)/height (m^2^).

### 2.2. Blood Biochemical Analyses

Fasting blood samples were drawn by venipuncture and centrifuged (1000× g, 15 min). Plasma and serum aliquots were kept at −20 °C until analyses. Plasma glucose, cholesterol, and triglycerides were determined by standard methods. Serum C-reactive protein (CRP) levels were determined by ELISA (CRP Human Instant ELISA, Ebioscience, San Diego, CA, USA), and malondialdehyde (MDA) by using the Byoxytech LPO-586 assay (Oxis International S.A., Paris, France) [26]. Serum leptin was determined by using the Human Leptin ELISA Development Kit 900-K90 (PeproTech Inc., Rocky Hill, NJ, USA) according to the manufacturer’s instructions.

### 2.3. Fecal Collection and Microbial Analysis

Participants were provided with a sterile container for fecal sample collection; after deposition samples were immediately frozen at −20 °C and transported to the laboratory. For analyses, samples were melted at room temperature (24 ± 2 °C), weighed, diluted 1/10 in sterile PBS, and homogenized (LabBlender 400 Stomacher, Seward Medical, London, UK) for 4 min; the DNA was extracted using the QIAamp DNA stool mini kit (Qiagen, Hilden, Germany) as described elsewhere [27]. Quantification of different bacterial populations, covering the major bacterial groups present in the human gut, was achieved in a 7500 Fast Real-Time PCR System (Applied Biosystems, Foster City, CA, USA) using SYBR Green PCR Master Mix (Applied Biosystems) [27] (Table 1). One microliter of template fecal DNA (~5 ng) and 0.2 μM of each primer were added to the 25 μL reaction mixture. PCR cycling consisted of an initial cycle of 95 °C 10 min, followed by 40 cycles of 95 °C 15 s, and 1 min at the appropriate primer−pair temperature. The number of cells was determined by comparing the Ct values obtained from a standard curve as previously described [27]. Fecal DNA extracts were analyzed and the mean quantity per gram of fecal wet weight was calculated for each bacterial group.

Major short chain fatty acid (SCFA), acetate, propionate, and butyrate were analyzed by gas chromatography from the supernatants of 1 mL of the homogenized feces as previously indicated [28]. A chromatograph 6890N (Agilent Technologies Inc., Palo Alto, CA, USA) connected to a mass spectrometry detector (MS) 5973N (Agilent Technologies) and a flame ionization detector (FID) was used for identification and quantification of SCFA, respectively, as described previously [29].

### 2.4. Statistical Analyses

Statistical analysis was performed using the IBM SPSS program version 24.0 (IBM SPSS, Inc., Chicago, IL, USA). Goodness of fit to the normal distribution was analyzed by means of the Kolmogorov−Smirnov test. When the distribution of variables was skewed, the natural logarithm of each value was used in the statistical test. For descriptive purposes, mean values are presented on untransformed variables. Differences in general and anthropometric characteristics, blood parameters, gut microbial groups, and fecal SCFA were assessed in accordance to tertiles of coffee intake through multivariate analyses adjusted by age, gender, BMI, and energy, based on the strong evidences linking these factors with human microbial composition. Pearson correlation was conducted to elucidate the interplay between caffeine and polyphenols from coffee and intestinal microbiota. The conventional probability value (0.05) for significance was used in the interpretation of results. Results obtained from the sample analysis were plotted using Microsoft Excel Software version 2016 (Microsoft Corporation, Redmon, Washington, USA). Data resulting from the Pearson correlation tests were plotted using GraphPad Prism version 8 for Windows (GraphPad Software, San Diego, CA, USA).

## 3. Results

The general characteristics of the sample are described in Table 2 according to the coffee tertiles. No statistically significant differences were found based on coffee consumption for any of the variables evaluated, with the exception of age, which was lower in subjects with the highest consumption of coffee (tertile 3). 

**Figure 1:**
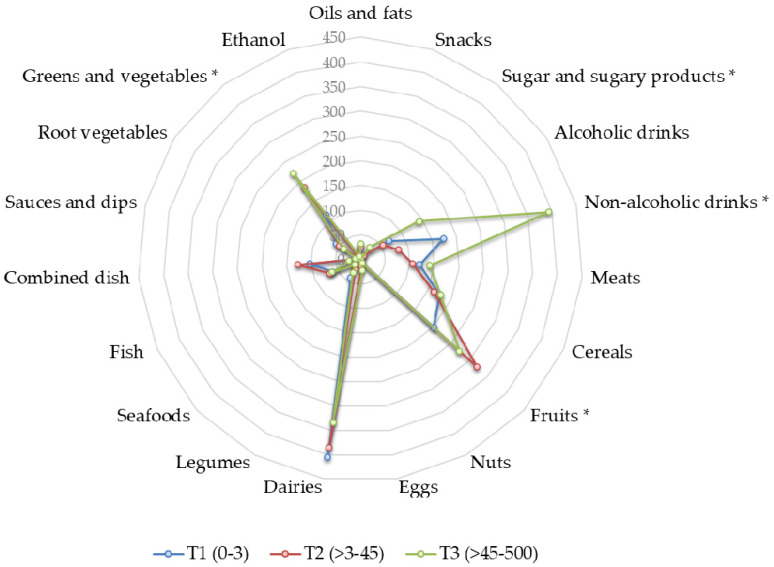
A radar plot representing differences in dietary patterns according to coffee consumption (mL/day) tertiles. * *p* ≤ 0.05.

The possible existence of a dietary pattern linked to coffee consumption was explored, as shown in Figure 1.

From the 19 items analyzed, only a moderate increase in the consumption of greens and vegetables was found across coffee tertiles, this being higher in tertile 3. As expected, since coffee is usually consumed with sugar, significant differences were also observed in the intake of non-alcoholic beverages and sugar products. In spite of this, the scarce differences found do not allow for a differential dietary pattern in the high-consumers group to be defined. When the average counts of the major gut microbial groups were analyzed, based on coffee consumption tertiles (Table 3), the sole difference observed was a significantly higher level of *Bacteroides-Prevotella-Porphyromonas* in tertile 3. Nevertheless, no differences were detected in fecal levels of SCFA according to coffee consumption, neither in the studied serum biomarkers, with the exception of MDA, an indirect biomarker of lipid peroxidation, whose concentration was lower in tertile 3. 

**Figure 2:**
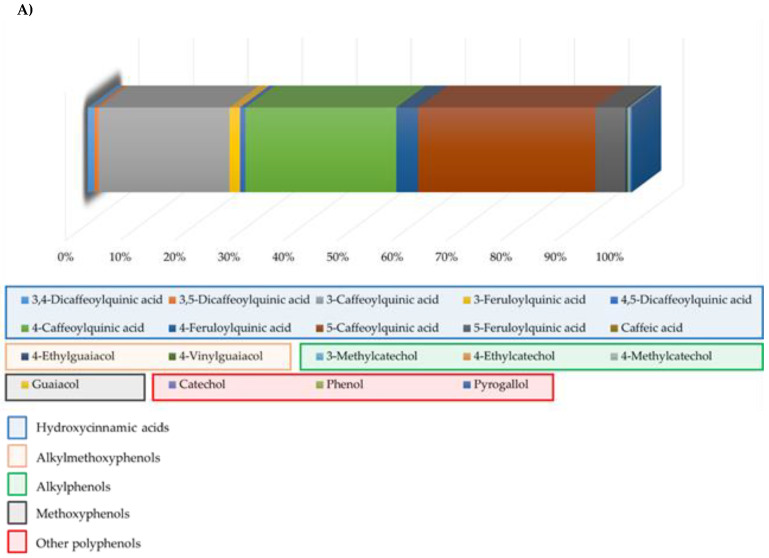

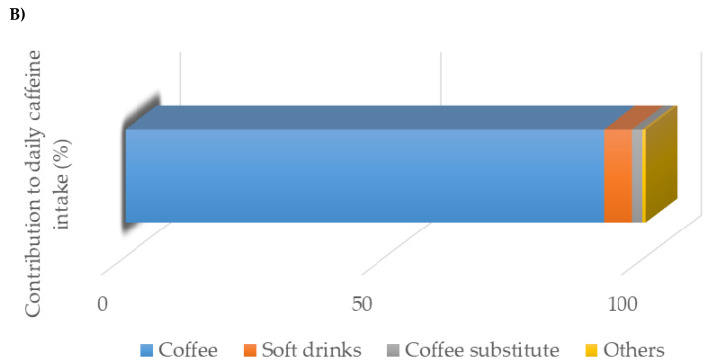
Representation of (**A**) the contribution of each coffee phenolic compound in the sample and (**B**) the dietary caffeine sources in the sample.

Coffee is a dietary source of various bioactive compounds including (poly)phenols and alkaloids (Figure 2). At the compound level, the major phenolic compounds provided by coffee were caffeoylquinic and feruloylquinic acids among hydroxycinnamics, and guaiacol from methoxyphenols (Figure 2A). As shown in Figure 2B, coffee was the major contributor to the intake of caffeine in the sample, explaining more than 90% of its consumption.

**Figure 3:**
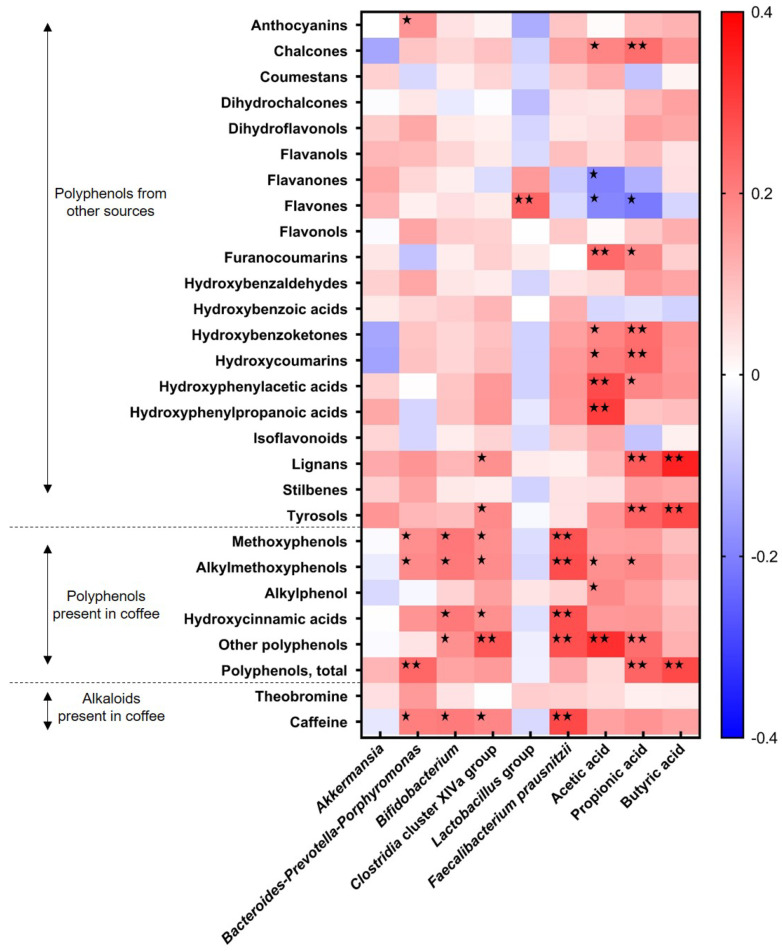
A heatmap showing Pearson correlations among intestinal microbial groups (Log n cells/gram feces), fecal short chain fatty acids (mM), polyphenol groups (mg/day), and alkaloids (mg/day), from coffee and other dietary sources. Columns correspond to major intestinal microbial groups and fecal SCFA; rows correspond to dietary polyphenols and alkaloids. Blue and red colors denote negative and positive association, respectively. The intensity of the colors represents the degree of association between variables. Asterisks indicate significant associations: * *p* ≤ 0.05; ** *p* ≤ 0.01.

Furthermore, the linear relationships between coffee derived dietary components and the microbiota was estimated through Pearson’s correlation test and are presented graphically in the heatmap of Figure 3. From the different phenolic compounds analyzed, those derived from coffee have shown the highest correlation with intestinal microbial groups together with caffeine. While metoxyphenols and alkylmethoxyphenols were correlated with the levels of the *Bacteroides–Prevotella–Porphyromonas* group with a *r* = 0.177 and 0.182, respectively, caffeine intake was directly associated with fecal *Bacteroides* levels (*r* = 0.200).

## 4. Discussion

Our results represent a first step in broadening the knowledge of the association between the regular intake of coffee and fecal microbiota in an apparently healthy human population, suggesting a possible implication of coffee phenolic compounds and caffeine in this relationship. 

The mean consumption of coffee is highly variable in the study sample, ranging between 0 and 500 mL/day, in a similar way to that observed in other European countries with a Mediterranean-type dietary pattern, such as Italy or Greece [30]. Given the absence of a reference value to establish coffee consumption levels, tertiles have been used to categorize the sample. The defined cut-off points are coherent from a methodological point of view, since they group non-coffee-consumers (0–3 mL/day) in tertile 1, moderate consumers (3–45 mL/day) in tertile 2, which could correspond to those subjects consuming a little cup of coffee per day of the so-called Italian coffee, and the tertile 3 of high consumers. Still, it must be taken into account that this tertile 3 has a lower mean intake of coffee than what has been reported in other countries, such as Germany [30]; therefore, our data may not be directly extrapolated to other countries with different trends in coffee consumption. It is also important to note that important differences in the coffee preparation procedures exist among different consumers and different countries. In our case, the mean coffee intake in tertile 3 is slightly lower than the range of 400–600 mL [9], associated with a protective effect against various pathologies [3,31]. Interestingly, in some studies the long-term consumption of seven cups of coffee per day has been associated with a reduction in the risk of metabolic syndrome, obesity, and type 2 diabetes [32,33]. However, since the coffee cup volume could vary from 150 mL to 300 mL among the studies included in the meta-analysis of Grosso et al. [32] indicated just before, extrapolations to our results are limited. 

In some impaired health conditions, alterations in the intestinal microbiota have been described, mainly a decrease in the abundance of *Bacteroides* and/or an increase in the Firmicutes/Bacteroidetes ratio [34,35,36]. In line with this, we have found higher fecal levels of the *Bacteroides-Prevotella-Porphyromonas* group in the high coffee consumers, supporting previous evidences in humans and animal models having higher fecal levels of these microorganisms in groups of better metabolic status [34,35,36]. It is tempting to speculate about this potential association; however, given the descriptive nature of this cross-sectional study, we cannot establish cause–effect relationships or directionality. Members of the phylum Bacteroidetes have been hypothesized to reduce intracellular oxygen levels, thus favoring the growing of anaerobic species which could promote the maintenance of intestinal balance, and they are identified as key glycan degrading bacteria [37,38] being more able to metabolize polyphenols than other groups such as Firmicutes. In this sense, coffee polyphenols explained a 20% of total polyphenol intake in the subjects with the highest consumption (tertile 3). Therefore, we hypothesized that at an equivalent total intake of polyphenols, the physiological effect of these compounds may differ among subjects depending on their dietary origin. It has been demonstrated that coffee-derived polyphenols were able to interact with intestinal bacteria in a bidirectional way: polyphenols may modify the gut environment [39], and/or they can be catabolized by intestinal bacteria converting them into a large variety of compounds with greater antioxidant activity than the compound of origin [40]. Despite some coffee-derived phenolic compounds, such as chlorogenic acid, having been associated in in vitro studies with important antioxidant and anti-inflammatory effects [41,42,43,44], we did not find differences in serum antioxidant capacity or in C-reactive protein, depending on the coffee consumption levels. Several factors may explain these results. Firstly, it is possible that the amount of coffee consumed in this sample was insufficient to observe a differential effect among tertiles, and secondly, subjects with low coffee consumption received polyphenols through other foodstuffs, thus ultimately achieving a similar polyphenol intake to the high coffee consumers (mean intake of 1604.4 and 1487.7 mg/day in T2 and T3 respectively, *p* = 0.533). Considering the high impact of phenolic compounds on gut microbial modulation, future human intervention studies analyzing the impact of coffee on fecal microbiota [9] should evaluate the intake of dietary and specific coffee polyphenols. 

Moreover, there is evidence from animal research showing that caffeine administration counteracts shifts in the ratio, Firmicutes/Bacteroides, resulting from a western diet [45]. Although we cannot attribute the observed differences in fecal microbial composition to a single compound, caffeine has been positively correlated in this work with most of the gut microbial groups analyzed. In turn, evidences of lower MDA concentrations in high coffee consumers may be in consonance with data in the literature describing the down-regulation effect of caffeine on lipid binding proteins and consequently in lipogenesis [46,47,48,49]. 

At the time of interpreting these results, the limited sample size should be considered. Nonetheless, we have found associations whose consistency and strength justify further research. Human experimentation in healthy subjects is limited by the logistical problems associated with carrying out direct measurements. Fecal SCFA accounts for only 5% to 10% of SCFA production that is not absorbed in the colon [50]. The FFQ is one of the most valid dietary tools in order to describe subjects’ regular dietary intake. However, to accurately quantify the coffee derived polyphenols, it would be advisable to register more detailed information about coffee such as the variety, the amount of powder used, ground grain size, and the final volume obtained [30]. Since we did not have individuals with daily intakes greater than 500 mL, it would be desirable in the future to be able to expand this group to deepen the association between this beverage and intestinal microbiota and oxidative stress, and to determine whether this would be dose dependent.

## 5. Conclusions

The interaction between coffee consumption, a modifiable factor, and intestinal bacteria may be useful for the development of dietary strategies in humans focused on diverse pathologies where the concentration of the *Bacteroides* group was altered. 

## Figures and Tables

**Table 1:** Primers and annealing temperatures used for the quantification of intestinal microbial groups by qPCR.

Microbial Group	Primer Sequence (5′-3′)	Tm. (°C)
*Akkermansia*	F: CAGCACGTGAAGGTGGGGAC R: CCTTGCGGTTGGCTTCAGAT	60
*Bacteroides-Prevotella-Porphyromonas*	F: GAGAGGAAGGTCCCCCAC R: CGCKACTTGGCTGGTTCAG	60
*Bifidobacterium*	F: GATTCTGGCTCAGGATGAACGC R: CTGATAGGACGCGACCCCAT	60
*Clostridia* cluster *XIVa* group	F: CGGTACCTGACTAAGAAGC R: AGTTTYATTCTTGCGAACG	55
*Faecalibacterium prausnitzii*	F: GGAGGAAGAAGGTCTTCGG R: AATTCCGCCTACCTCTGCACT	60
*Lactobacillus* group	F: AGCAGTAGGGAATCTTCCA R: CATGGAGTTCCACTGTCCTC	60

Adapted from Reference [28].

**Table 2:** General characteristics of the study sample according to coffee consumption tertiles.

Characteristic	Coffee (mL/day)		
	T1 (0–3) (*n* = *49*)	T2 (>3–45) (*n* = *49*)	T3 (>45–500) (*n* = *49*)
Age (years)	58.8 ± 18.62 _a_	67.57 ± 14.77 _b_	47.10 ± 10.86 _c_
Gender (% female)	69%	71%	71%
BMI (kg/m^2^)	28.08 ± 4.52 _a_	27.32 ± 3.55 _a_	26.73 ± 5.16 _a_
Sleep duration (h/day)	6.78 ± 1.06 _a_	6.73 ± 1.07 _a_	7.00 ± 1.31 _a_
Energy intake (Kcal/day)	1906.93 ± 494.27 _a_	1776.89 ± 531.64 _a_	2040.23 ± 622.47 _a_
Coffee consumption (mL/day)	0.15 ± 0.59 _a_	27.53 ± 11.14 _b_	151.84 ± 92.10 _c_
Tobacco user (%)	25%	28%	25%
Depositions (nº/week)	8.89 ± 6.26 _a_	6.49 ± 2.68 _b_	7.74 ± 3.84 _a,b_

All values are shown as mean ± standard deviation (SD). Values in the same row showing a different subscript present a statistically significant difference (*p* ≤ 0.05). Tobacco user refers people with smoking-habit at the time of the study.

**Table 3:** Differences in gut microbiota composition, fecal short chain fatty acids concentration (SCFA), and serum markers according to coffee consumption tertiles.

	Coffee (mL/day)		
	T1 (0–3)	T2 (>3–45)	T3(>45–500)
Model 1. Microbial group (Log n cells/gram feces) (n, 138) *			
*Akkermansia*	5.70 ± 0.40 _a_	5.76 ± 0.33 _a_	6.30 ± 0.36 _a_
*Bacteroides-Prevotella-Porphyromonas*	8.03 ± 0.27 _a_	8.74 ± 0.27 _a,b_	9.14 ± 0.30 _b_
*Bifidobacterium*	7.61 ± 0.26 _a_	7.73 ± 0.26 _a_	8.19 ± 0.28 _a_
*Clostridia* cluster XIVa group	7.48 ± 0.29 _a_	7.49 ± 0.29 _a_	7.50 ± 0.31 _a_
*Lactobacillus* group	6.28 ± 0.27 _a_	5.97 ± 0.27 _a_	5.97 ± 0.29 _a_
*Faecalibacterium prausnitzii*	7.10 ± 0.17 _a_	7.29 ± 0.17 _a_	7.51 ± 0.19 _a_
Model 2. Fecal SCFA concentration (mM) (n, 132) *			
Acetic acid	36.77 ± 2.51 _a_	36.80 ± 2.48 _a_	33.99 ± 2.58 _a_
Propionic acid	12.55 ± 1.09 _a_	13.97 ± 1.08 _a_	12.56± 1.12 _a_
Butyric acid	10.17 ± 1.18 _a_	10.90 ± 1.17 _a_	10.10 ± 1.21 _a_
Model 3. Blood parameters *			
Serum MDA (µM) (n,102)	2.51 ± 0.11 _a_	2.28 ± 0.07 _a,b_	1.89 ± 0.20 _b_
C reactive protein (mg/L) (n,108)	1.37 ± 0.24 _a_	1.27 ± 0.17 _a_	0.69 ± 0.46 _a_
Leptin (ng/mL) (n,102)	11.05 ± 1.21 _a_	11.15 ± 0.85 _a_	8.34 ± 2.34 _a_
LDL–HDL ratio (n,125)	2.51 ± 0.18 _a_	2.86 ± 0.13 _a_	2.85 ± 0.35 _a_
Triglycerides (mg/dL) (n,125)	121.70 ± 11.12 _a_	122.08 ± 7.80 _a_	103.14 ± 21.44 _a_
Glucose (mg/dL) (n,125)	100.12 ± 5.62 _a_	103.05 ± 3.94 _a_	103.73 ± 10.84 _a_
Antioxidant capacity (mM) (n,72)	0.36 ± 0.02 _a_	0.34 ± 0.01 _a_	0.34 ± 0.04 _a_

* Results obtained from multivariate analyses adjusted by age, gender, BMI, and energy. Values in the same row showing different subscripts present a statistically significant difference; (*p* ≤ 0.05). MDA, malondialdehyde; LDL, low density lipoprotein; HDL, high density lipoprotein.
